# Tamoxifen Enhances the Hsp90 Molecular Chaperone ATPase Activity

**DOI:** 10.1371/journal.pone.0009934

**Published:** 2010-04-01

**Authors:** Rongmin Zhao, Elisa Leung, Stefan Grüner, Matthieu Schapira, Walid A. Houry

**Affiliations:** 1 Department of Biochemistry, University of Toronto, Toronto, Ontario, Canada; 2 Structural Genomics Consortium and Department of Pharmacology, University of Toronto, Toronto, Ontario, Canada; Monash University, Australia

## Abstract

**Background:**

Hsp90 is an essential molecular chaperone that is also a novel anti-cancer drug target. There is growing interest in developing new drugs that modulate Hsp90 activity.

**Methodology/Principal Findings:**

Using a virtual screening approach, 4-hydroxytamoxifen, the active metabolite of the anti-estrogen drug tamoxifen, was identified as a putative Hsp90 ligand. Surprisingly, while all drugs targeting Hsp90 inhibit the chaperone ATPase activity, it was found experimentally that 4-hydroxytamoxifen and tamoxifen enhance rather than inhibit Hsp90 ATPase.

**Conclusions/Significance:**

Hence, tamoxifen and its metabolite are the first members of a new pharmacological class of Hsp90 activators.

## Introduction

Hsp90 is a ubiquitous ATP-dependent dimeric molecular chaperone that plays a central role in cellular signalling pathways since it is essential for maintaining the activity of several signalling proteins including steroid hormone receptors and protein kinases. In eukaryotes, cytoplasmic Hsp90 is absolutely essential for cell viability under all growth conditions [1], [2]. Hsp90s typically function as part of large complexes that include other chaperones and essential cofactors regulating Hsp90 function. It is thought that different cofactors target Hsp90 to different sets of substrates. However, the mechanism of Hsp90 function remains poorly understood.

The broad effect of Hsp90 on protein cellular homeostasis arises from the large number of client substrates that this chaperone acts upon. For example, we recently demonstrated that about 10% of the yeast proteome genetically or physically interacts with Hsp90 [3]. In mammalian cells, more than one hundred Hsp90 substrates have been identified including a large number of transcription factors and protein kinases such as glucocorticoid receptor, p53 mutant, vSrc, Hck, CFTR, and many others [4].

Hsp90 is currently a novel anti-cancer drug target [5], [6]. The inhibition of Hsp90 activity typically results in the degradation of Hsp90 client substrates and, consequently, in the blocking of multiple cellular pathways [7]. This seems to affect tumour progression [8]. Several Hsp90 inhibitors have been identified from natural products and some have also been chemically synthesized. For example, fungal antibiotics belonging to the benzoquinone ansamycin family, such as geldanamycin, herbimycin A, and macbecin, were found to specifically bind to Hsp90. It has been shown that geldanamycin directly binds to the ATP-binding pocket in the N-terminal domain of Hsp90 [9], [10] and blocks the binding of nucleotides to Hsp90. A geldanamycin derivative, 17-(allylamino)-17-demethoxygeldanamycin (17-AAG), is now progressing to phase II clinical trials [11], [12], [13], [14]. Other natural product Hsp90 inhibitors include the macrolide radicicol and its derivatives [15]. Synthetic small-molecule inhibitors of Hsp90 have also been designed based on the available X-ray structures of the chaperone. One such class of Hsp90 inhibitors are the purine scaffold derivatives (such as PU3) [16], while another such synthetic inhibitors are the pyrazole resorcinols [17].

Hence, there is growing interest in developing new drugs that modulate Hsp90 activity. While Hsp90 inhibition has been clearly established as a valid entry point for clinical intervention in oncology, the therapeutic relevance of Hsp90 activation in other disease areas remains unexplored. In this study, we report the unexpected identification of small molecule activators of Hsp90. Such activators of Hsp90 ATPase have not been described before and will be useful to investigate the pharmacology of Hsp90 activation.

## Materials and Methods

### Docking Procedure

A library of 4403 pockets co-crystallized with ligands of molecular weight between 100 to 500 Da having a volume larger than 200 Å^3^ and a resolution better than 2.5 Å was extracted from the Protein Data Bank (PDB) using the IcmPocketFinder macro of the ICM computational chemistry platform (Molsoft LLC, CA), as described elsewhere [18]. The library contained 16 Hsp90 pockets and 29 pockets from other chaperones (Table S1). Each site was automatically reformatted into a dock-ready grid potential that accounts for van der Waals shape, hydrophobicity profile, and hydrogen-bond network. The flexible ligand was docked with ICM to each grid by a Monte Carlo energy minimization in the internal coordinates space and assigned a score based on the quality of the fit, including the entropy of the system and Poisson electrostatics [19]. ICM docking thoroughness was set to 1 and default virtual screening parameters of version 3.6-1d of the software were used. The simulation takes on average 45 seconds per receptor per CPU.

### Protein Expression and Purification

*Escherichia coli* ClpX [20] and human Hsp70 [21] were purified as previously described. *Saccharomyces cerevisiae* (yeast) Hsp82, different domains of yeast Hsp82, and human Hsp90α were expressed in *E. coli* strain BL21-CodonPlus (DE3)-RIL (Stratagene) from plasmid pProEX HTa with a His_6_-tag followed by a tobacco etch virus (TEV) protease cleavage site fused at the N-terminus of the protein. Protein expression was induced with 1 mM IPTG. Cells were broken using the French Press and were then purified on Ni-NTA resin (QIAGEN) followed by size-exclusion chromatography using Superdex 200 (GE Healthcare) column. All proteins were purified in their native state. Proteins were stored in buffer A (25 mM TrisHCl, pH 7.5, 150 mM KCl, 10% glycerol, and 0.5 mM DTT).

The untagged *E. coli* HtpG was expressed in *E. coli* BL21 (DE3) transformed with plasmid pHB40P (a gift from Dr. David Agard). Cells were lysed by sonication and the protein was precipitated by (NH_4_)_2_SO_4_ at a final concentration of 75%. The pellet was resuspended and applied onto Q-Sepharose (GE Healthcare). The HtpG eluant, at about 500 mM KCl, was applied on Superdex 200 size exclusion column and then stored in buffer A.

### ATPase Activity Assay

ATPase activity of Hsp90s, ClpX, and Hsp70 were measured using the coupled LDH/NADH method as described [22]. Briefly, the reaction mixture was set up in a final volume of 500 µL containing 25 mM HEPES, 5 mM MgCl_2_, 3 µM Hsp90 or 0.6 µM ClpX or 1.4 µM Hsp70 (monomer concentration), 5 mM ATP, 0.2 mM NADH, 3 mM phosphoenolpyruvate, 15.6 U pyruvate kinase (Sigma), 24.5 U lactate dehydrogenase (Sigma), 0.03% Tween 20, and 10% glycerol. For experiments in high salt concentration, KCl was included in the reaction mix at a final concentration of 400 mM, otherwise, 40 mM KCl was included. The decrease in NADH absorbance at 340 nm was recorded continuously for 40 minutes using a Cary 300 Bio UV-VIS spectrophotometer (Varian).

Before starting the reaction, the mixture without the ATPase was incubated at 37°C for 2 minutes and then NADH absorbance was recorded for 3 minutes and used as background to account for spontaneous ATP hydrolysis. The ATPase was then added and ATP hydrolysis was monitored by the decrease in NADH absorbance. Tamoxifen (Sigma), 4-hydroxytamoxifen (Sigma), raloxifene (Sigma), or geldanamycin (National Institute of Health) dissolved in DMSO was subsequently added to the reaction. The purity of tamoxifen and 4-hydroxytamoxifen (4-OHT) was judged to be >98% based on mass spectrometry analysis of the samples. The specific activity of the ATPase was calculated as described [22].

### Surface Plasmon Resonance (SPR) measurements

SPR measurements were conducted on a BiacoreX instrument (GE Healthcare) at 25°C. Yeast Hsp82 was immobilized to one of two flow cells on CM5 chips (GE Healthcare) using the Biacore amine coupling kit (GE Healthcare) following the manufacturer's protocols. The reference flow cell was sham activated and deactivated without protein immobilization. Immobilizations were performed in buffer B (10 mM HEPES, pH 7.5, 150 mM KCl, 3 mM EDTA, and 0.005% P20 surfactant). After surface activation, 10,000–15,000 Response Units (RU) of immobilized ligand was typically obtained with a 30 µL injection of 50 µg/mL yeast Hsp82 or Hsp82 N-domain (Hsp82N) in 10 mM sodium acetate, pH 4.5 over the activated flow cell.

Direct binding experiments were performed at a flow rate of 20 µL/min in buffer C (10 mM HEPES, pH 7.5, 150 mM KCl, 10 mM MgCl_2_, 3 mM EDTA, and 0.005% P20 surfactant). Various concentrations of ADP, raloxifene, or 4-OHT analyte were injected over yeast Hsp82 coupled biosensor chip surfaces. Each 60 seconds injection of analyte was followed by 60 seconds of running buffer flow before the chip and sample loop were washed using high flow rate, after which, the sensorgram signal returned to baseline. The sensorgrams in the reference surface were subtracted from the corresponding sensorgrams in the protein-immobilized flow cell to remove the effect of nonspecific binding to the chip surface and the bulk effect from the buffer. Since the high refractive index of the DMSO in the sample combined with the high density of immobilized protein on the ligand surface resulted in an excluded volume effect, each injection was double referenced with a second injection of equivalent buffer composition minus analyte. For the direct binding experiments, each injection contained 2% DMSO. The steady state responses were plotted versus the corresponding analyte concentrations and fit to a one-site Langmuir binding model by nonlinear regression using the BiaEvaluation 4.1 software (GE Healthcare).

As a variation to the above-described equilibrium binding experiments, binding curves for ADP or ATP to chip-immobilized yeast Hsp82 or Hsp82N were obtained in the presence of raloxifene and 4-OHT. The running buffer used in these experiments mimicked the high salt buffer used in the ATPase assays, buffer D (25 mM HEPES, pH 7.5, 400 mM KCl, 5 mM MgCl_2_, and 10% DMSO) with 25 µM of 4-OHT or raloxifene. The injection phase was 120 seconds followed by 60 seconds of buffer flow before the cells were washed at high flow rate.

## Results

### Effect of 4-hydroxytamoxifen (4-OHT) on Hsp90 ATPase activity

**Figure 1:**
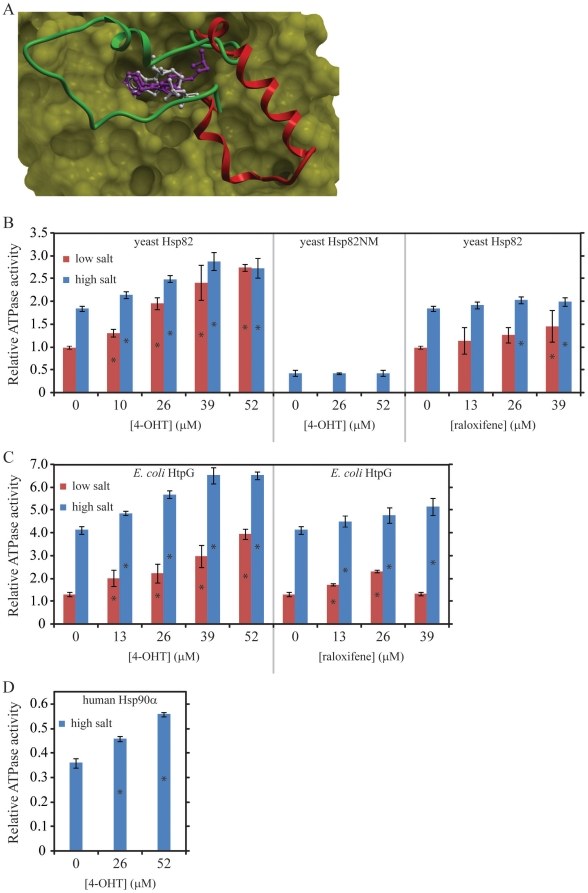
4-OHT Activates Hsp90 ATPase. (**A**) Conformation of 4-OHT (magenta) docked onto the crystal structure of the N-terminal ATP-binding domain of human Hsp90α (yellow mesh, pdb code 1uy8). 4-OHT occupies the nucleotide binding pocket as illustrated by the superimposed structure of human Hsp90α N-terminal domain (not shown for clarity–pdb code 1byq, backbone RMSD 1.45 Å) co-crystallized with ADP (white). Unlike ADP, the 4-OHT aliphatic chain extends deep into the pocket, and makes multiple hydrophobic contacts with the lid which acts as a structural switch between the active (green–pdb code 2cg9) and inactive (red–pdb code 1uy8) conformation of the enzyme. (**B–D**) The effects of 4-OHT and raloxifene on Hsp90 ATPase activity are shown in low salt (40 mM KCl, red) and high salt (400 mM KCl, blue) buffer. All ATPase activities refer to the ATPase inhibited by 20 µM geldanamycin (refer to Materials and Methods) and are relative to that of yeast Hsp82, which has a measured geldanamycin-inhibited ATPase activity of 4.85 (±0.19) pmol Pi min^−1^ µg^−1^. Data shown are averages of three to five repeats. Error bars refer to the standard deviation. Stars on selected bars indicate that the average value obtained for that experiment is statistically different from the average obtained in the absence of the drug using the Z-test at the 95% confidence limit.

The project initially started with the intent of identifying unexpected targets of the anti-estrogen drug tamoxifen (TAM). Hence, 4-hydroxytamoxifen (4-OHT), the active metabolite of TAM, was docked to the crystal structure of over 4000 druggable pockets extracted from the protein data bank using a virtual screening approach [19]. Each structure was assigned a score based on the quality of the fit (Table S1). The top 15 scoring hits were 12 estrogen receptor structures and 3 Hsp90 structures (pdb codes 1uyf, 1uy8, and 2ior–see Table S1). The hits to estrogen receptors are expected since TAM competes with estrogen for binding to estrogen receptors, and is typically used in the treatment of advanced breast cancer in women whose tumors are estrogen-dependent [23]. However, the hits to Hsp90 were unexpected (Figure 1A) and suggested a novel activity for TAM and its derivatives. It should be pointed out that, even though 4-OHT docked well to the Hsp90 ATP pocket, no similarity was identified between the structural chemistry of this site and of the ligand binding pocket of the estrogen receptor using the online server SuMo [24]. As a negative control, celebrex, another unrelated drug, was docked to the same collection of pockets. While cyclooxygenase 2, the known target of celebrex, was ranked number 1, the first Hsp90 structure ranked 132 with a bad score (Table S1).

**Figure 2:**
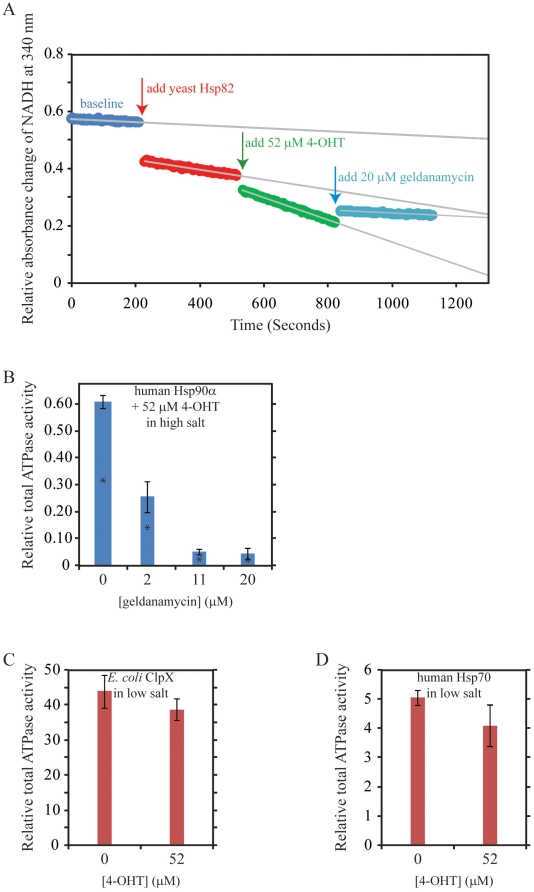
Control Experiments for the Effect of 4-OHT on Hsp90 ATPase. (**A**) Raw ATPase data showing the effect of 4-OHT on yeast Hsp82 as monitored by the decrease in NADH absorbance. (**B**) Effect of geldanamycin on human Hsp90α ATPase activity in the presence of 52 µM 4-OHT in high salt buffer. (**C**) Effect of 4-OHT on the ATPase activity of *E. coli* ClpX in low salt buffer. (**D**) Effect of 4-OHT on the ATPase activity of human Hsp70 in low salt buffer. All ATPase activities given refer to total activity and not to the geldanamycin-inhibited ATPase. They are given relative to the geldanamycin-inhibited ATPase activity of yeast Hsp82 [4.85 (±0.19) pmol Pi min^−1^ µg^−1^]. Data shown are averages of three to five repeats. Error bars refer to the standard deviation. Stars on selected bars indicate that the average value obtained for that experiment is statistically different from the average obtained in the absence of the drug using the Z-test at the 95% confidence limit.

The effect of 4-OHT on the ATPase activity of Hsp90 was then measured at low salt (40 mM KCl) and high salt concentrations (400 mM KCl) using different forms of Hsp90. Salt alone enhanced the ATPase activity of *Saccharomyces cerevisiae* (yeast) and *E. coli* Hsp90 by a factor of about 2 and 4, respectively (Figure 1B, C). This is consistent with previous reports [25]. For human Hsp90, reliable data was only obtained at high salt concentration since this chaperone has very low ATPase activity. To our surprise, we found that 4-OHT enhanced, rather than inhibited, the ATPase activity of yeast, *E. coli*, and human Hsp90 (Figures 1B–D and Figure 2A). Such an enhancement was also observed for TAM (data not shown).

At low salt concentration, the enhancement by 52 µM 4-OHT was about 3 fold, while, at high salt concentration, the enhancement was about 1.5 fold for all three forms of Hsp90 (Figure 1B–D). The ATPase activity measured was inhibited by geldanamycin (Figure 2A,B), clearly indicating that the activity arises from Hsp90. Another selective estrogen receptor modulator, raloxifene, did not enhance Hsp90 ATPase activity as much as 4-OHT (Figure 1B, C). For example, in low salt buffer, 39 µM raloxifene enhanced Hsp90 ATPase by 1.46 (±0.35) fold, while 39 µM 4-OHT enhanced the chaperone ATPase by 2.42 (±0.38) (Figure 1B). Raloxifene docked well to one of the Hsp90 structures (pdb code 1uyf), but not to the best 2 scoring structures for tamoxifen (pdb codes 2ior and 1uy8). This difference in docking results between tamoxifen and raloxifene may reflect the observed difference in their ability to activate HSP90, although it might just be a result of the limits of docking simulations.

The enhancement of Hsp90 ATPase activity by 4-OHT requires the full length chaperone, since no such effect was observed when using a truncated version of yeast Hsp90 containing only the N-terminal ATP-binding domain and the middle domain of the chaperone (Figure 1B). This construct has about 40% of the ATPase activity of the full length chaperone in the high salt buffer. Furthermore, 4-OHT did not affect the ATPase activity (maybe caused a slight inhibition) of other unrelated chaperones such as *E. coli* ClpX and human Hsp70 (Figure 2C,D).

### Binding of 4-OHT to Hsp82

**Figure 3:**
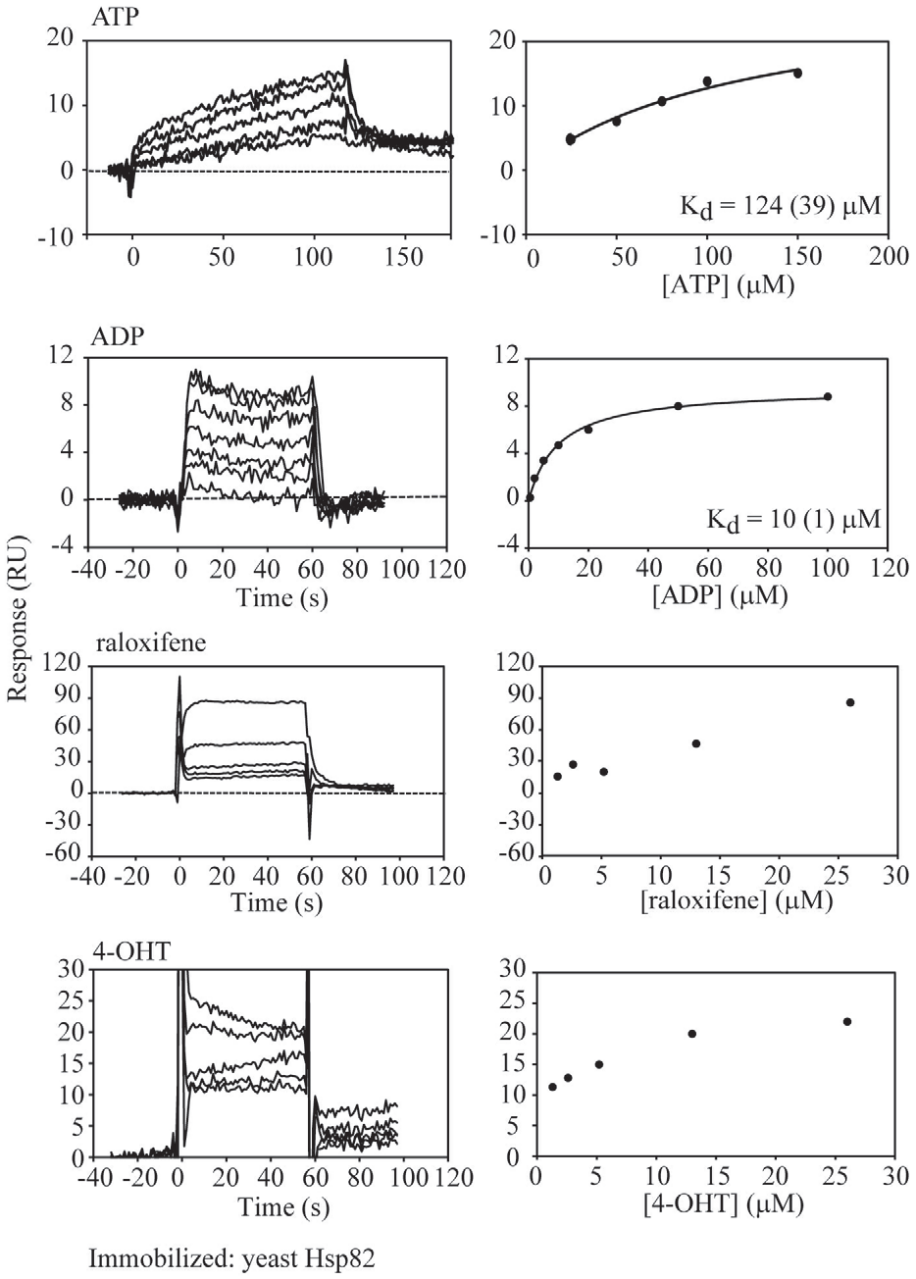
SPR Measurements for ATP, ADP, Raloxifene, and 4-OHT Binding to Yeast Hsp82 Immobilized on Biosensor Chip. Various concentrations of ATP, ADP, raloxifene, and 4-OHT were injected over a surface immobilized with yeast Hsp82. The sensorgrams shown have been double referenced. The steady state responses were fit, where possible, using nonlinear regression to a single class of binding site model to obtain the K_d_ values indicated. Numbers in parenthesis give the standard errors on the K_d_ obtained from the fits.

**Figure 4:**
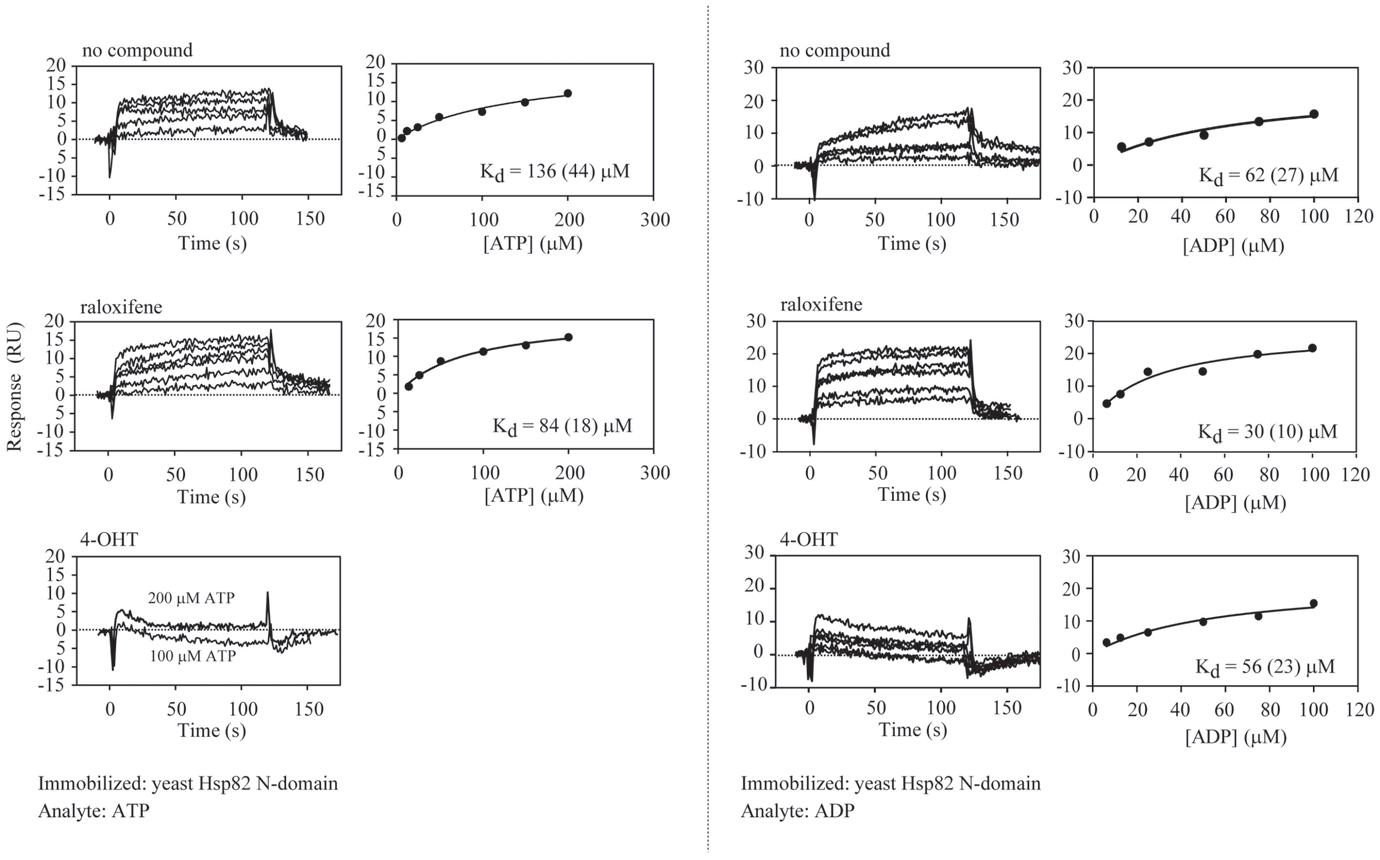
Effect of 4-OHT on Nucleotide Binding to Yeast Hsp82 N-Domain Immobilized on Biosensor Chip. Various concentrations of ATP or ADP were injected over a biosensor chip with immobilized Hsp82N using running buffer containing no drug (top panels), 25 µM raloxifene (middle panels), or 25 µM 4-OHT (bottom panels). The sensorgrams shown have been double referenced. The steady state responses were fit as in Figure 3.

The observed enhancement of Hsp90 ATPase activity by 4-OHT in our experiments seems to contradict the docking model (Figure 1A) in which the drug molecule occupies the ATP-binding pocket, and should presumably compete with ATP-binding to Hsp90. Hence, we next attempted to experimentally demonstrate that 4-OHT binds to the N-terminus of Hsp82 as predicted by the docking approach. To this end, we carried out surface plasmon resonance (SPR) experiments using the Biacore system. The binding constants obtained for the direct binding of ATP or ADP to full length yeast Hsp82 (Figure 3) or Hsp82N (Figure 4) immobilized on the biosensor chip were in general agreement with those published in the literature (refer for example to reference [10]). It is generally observed that the binding of ATP to Hsp82 or Hsp82N is weaker than that of ADP. Both the titrations of raloxifene and 4-OHT were problematic (Figure 3), since the high refractive index of DMSO in the samples gave rise to artifacts from the differences between the running buffer and the injected sample. The titration of raloxifene yielded data which did not reach saturation indicating unspecific binding. On the other hand, the titration of 4-OHT, while also quite noisy, did reach saturation, although the fits did not provide a reliable value for the K_d_. The K_d_ is estimated to be between 10 and 100 µM. The same experiments were done using chip-immobilized yeast Hsp82N with similar results (data not shown). Binding experiments were also attempted using isothermal titration calorimetry, however, again we encountered problems with low signal and high noise.

In another approach to demonstrate that 4-OHT can directly compete with nucleotides for binding to Hsp82N, the SPR experiments were repeated but raloxifene or 4-OHT was included in the running buffer at 25 µM. As shown in Figure 4, raloxifene did not compete appreciably with ATP or ADP binding to Hsp82N. However, when 4-OHT was included with the running buffer, no net binding response was observed up to 200 µM ATP (Figure 4). On the other hand, the binding of ADP to Hsp82N was not significantly affected by the presence of 25 µM 4-OHT. These results seem to suggest that the binding of 4-OHT to Hsp90 is stronger than ATP but weaker than ADP. Hence, we estimate the K_d_ for binding of 4-OHT to Hsp82 to be of the order of 60–100 µM.

## Discussion

The observation that 4-OHT activates rather than inhibits Hsp90 ATPase, even though it was initially docked onto the N-terminal ATP-binding pocket of the chaperone (Figure 1A) is an unexpected finding. The SPR experiments of Figures 3 and 4 seem to suggest that 25 µM 4-OHT can compete with up to 200 µM ATP for binding to Hsp82 N-domain, but not with ADP. The results do not formally exclude the possibility that 4-OHT might bind at a site in Hsp82N different from that of the ATP pocket, which would cause an allosteric inhibition for the binding of ATP in the nucleotide pocket.

The ATPase assays were carried out using 4-OHT concentrations up to 52 µM and ATP concentration of 5 mM (Figures 1 and 2), while Hsp90 concentration is 3 µM (see Materials and Methods). Since the K_d_ of 4-OHT and nucleotide binding to Hsp90 are all in the µM range, (Figure 3 and 4), then, under the ATPase assay condition, we expect nucleotide to be preferentially bound by Hsp90 by a factor of approximately 1000 to 1 over 4-OHT. To reconcile the observation that 4-OHT binds at or near the ATP pocket but at the same time enhances the ATPase activity of Hsp90 when present at 1000 fold lower concentrations than that of ATP, we propose that, 4-OHT might affect the kinetics of ATP hydrolysis and ADP or Pi release. This seems to be suggested by the results of the experiments of Figure 4. The validation of this proposal will require detailed kinetic, mutational, and structural analyses of drug binding to Hsp90. For example, based on the observations made, we expect that increasing the concentration of 4-OHT to mM concentrations in the ATPase assay should eventually inhibit the ATPase activity of Hsp90. Unfortunately, 4-OHT is not an ideal drug for such studies due to its limited solubility and tendency to precipitate at concentrations above 52 µM used in our assays (Figures 1 and 2).

To our knowledge, 4-OHT is the first small molecule of a new pharmacological class of Hsp90 activators. Although this off-target effect of 4-OHT on Hsp90 is only observed at micromolar drug concentrations, it is probable that 4-OHT can be chemically modified to become a more potent activator of Hsp90. The structural mechanism of pharmacological activation of Hsp90 and its potential translational applications deserve further investigation.

## Supporting Information

Table S1 The ICM docking score of 4-hydroxy tamoxifen and celebrex against all 4403 pockets extracted from the PDB are shown. The lowest scores are the best. ICM user's manual recommends to discard docking results scoring higher than -32.
(1.44 MB XLS)

## References

[1] Borkovich KA, Farrelly FW, Finkelstein DB, Taulien J, Lindquist S (1989). hsp82 is an essential protein that is required in higher concentrations for growth of cells at higher temperatures.. Mol Cell Biol.

[2] Cutforth T, Rubin GM (1994). Mutations in Hsp83 and cdc37 impair signaling by the sevenless receptor tyrosine kinase in Drosophila.. Cell.

[3] Zhao R, Davey M, Hsu YC, Kaplanek P, Tong A (2005). Navigating the chaperone network: an integrative map of physical and genetic interactions mediated by the hsp90 chaperone.. Cell.

[4] Pratt WB, Toft DO (2003). Regulation of signaling protein function and trafficking by the hsp90/hsp70-based chaperone machinery.. Exp Biol Med (Maywood).

[5] Neckers L (2002). Hsp90 inhibitors as novel cancer chemotherapeutic agents.. Trends Mol Med.

[6] Pearl LH, Prodromou C, Workman P (2008). The Hsp90 molecular chaperone: an open and shut case for treatment.. Biochem J.

[7] Workman P, Burrows F, Neckers L, Rosen N (2007). Drugging the cancer chaperone HSP90: combinatorial therapeutic exploitation of oncogene addiction and tumor stress.. Ann N Y Acad Sci.

[8] Pearl LH, Prodromou C (2006). Structure and mechanism of the Hsp90 molecular chaperone machinery.. Annual Review of Biochemistry.

[9] Stebbins CE, Russo AA, Schneider C, Rosen N, Hartl FU (1997). Crystal structure of an Hsp90-geldanamycin complex: targeting of a protein chaperone by an antitumor agent.. Cell.

[10] Prodromou C, Roe SM, O'Brien R, Ladbury JE, Piper PW (1997). Identification and structural characterization of the ATP/ADP-binding site in the Hsp90 molecular chaperone.. Cell.

[11] Maloney A, Workman P (2002). HSP90 as a new therapeutic target for cancer therapy: the story unfolds.. Expert Opin Biol Ther.

[12] Dunn FB (2002). Heat shock protein inhibitor shows antitumor activity.. J Natl Cancer Inst.

[13] Workman P (2002). Pharmacogenomics in cancer drug discovery and development: inhibitors of the Hsp90 molecular chaperone.. Cancer Detect Prev.

[14] Modi S, Stopeck AT, Gordon MS, Mendelson D, Solit DB (2007). Combination of trastuzumab and tanespimycin (17-AAG, KOS-953) is safe and active in trastuzumab-refractory HER-2 overexpressing breast cancer: a phase I dose-escalation study.. J Clin Oncol.

[15] Agatsuma T, Ogawa H, Akasaka K, Asai A, Yamashita Y (2002). Halohydrin and oxime derivatives of radicicol: synthesis and antitumor activities.. Bioorg Med Chem.

[16] Vilenchik M, Solit D, Basso A, Huezo H, Lucas B (2004). Targeting wide-range oncogenic transformation via PU24FCl, a specific inhibitor of tumor Hsp90.. Chem Biol.

[17] Eccles SA, Massey A, Raynaud FI, Sharp SY, Box G (2008). NVP-AUY922: a novel heat shock protein 90 inhibitor active against xenograft tumor growth, angiogenesis, and metastasis.. Cancer Res.

[18] An J, Totrov M, Abagyan R (2004). Comprehensive identification of “druggable” protein ligand binding sites.. Genome Inform.

[19] Totrov M, Abagyan R (1997). Flexible protein-ligand docking by global energy optimization in internal coordinates.. Proteins.

[20] Wojtyra UA, Thibault G, Tuite A, Houry WA (2003). The N-terminal zinc binding domain of ClpX is a dimerization domain that modulates the chaperone function.. J Biol Chem.

[21] Mosser DD, Ho S, Glover JR (2004). Saccharomyces cerevisiae Hsp104 enhances the chaperone capacity of human cells and inhibits heat stress-induced proapoptotic signaling.. Biochemistry.

[22] Norby JG (1988). Coupled assay of Na+,K+−ATPase activity.. Methods Enzymol.

[23] Jordan VC (2006). Tamoxifen (ICI46,474) as a targeted therapy to treat and prevent breast cancer.. Br J Pharmacol.

[24] Jambon M, Imberty A, Deleage G, Geourjon C (2003). A new bioinformatic approach to detect common 3D sites in protein structures.. Proteins.

[25] Panaretou B, Siligardi G, Meyer P, Maloney A, Sullivan JK (2002). Activation of the ATPase activity of hsp90 by the stress-regulated cochaperone aha1.. Mol Cell.

